# A Structural View on ESCRT-Mediated Abscission

**DOI:** 10.3389/fcell.2020.586880

**Published:** 2020-11-09

**Authors:** Péter Horváth, Thomas Müller-Reichert

**Affiliations:** Experimental Center, Faculty of Medicine Carl Gustav Carus, Technische Universität Dresden, Dresden, Germany

**Keywords:** endosomal sorting complexes required for transport (ESCRT), cytokinesis, abscission, microtubules, membranes, electron microscopy, electron tomography, 3D reconstruction

## Abstract

The endosomal sorting complex required for transport (ESCRT) mediates cellular processes that are related to membrane remodeling, such as multivesicular body (MVB) formation, viral budding and cytokinesis. Abscission is the final stage of cytokinesis that results in the physical separation of the newly formed two daughter cells. Although abscission has been investigated for decades, there are still fundamental open questions related to the spatio-temporal organization of the molecular machinery involved in this process. Reviewing knowledge obtained from *in vitro* as well as *in vivo* experiments, we give a brief overview on the role of ESCRT components in abscission mainly focussing on mammalian cells.

## Introduction

Cytokinesis, the physical separation of newly formed daughter cells, is a very sophisticated process that is precisely coordinated and controlled by a complex molecular machinery. Proteome analysis revealed 577 proteins in purified intact midbodies of Chinese hamster ovary (CHO) cells (Skop et al., 2004) and 1730 proteins in the midbody interactome of HeLa cells (Capalbo et al., 2019). In addition, the midbody remnant in HeLa cells consisted of 1732 proteins (Addi et al., 2020). To systematically analyze cytokinesis, it is practical to break this complex process into the following discrete steps: assembly and ingression of the contractile actin ring to achieve a primary constriction leading to the formation of the intercellular bridge; rearrangement of microtubules during bridge formation; and secondary constriction of the intercellular bridge culminating in abscission (Bhutta et al., 2014). There are distinct processes controlling the order of events in abscission of animal cells: formation, stabilization and severing of microtubules at the intercellular bridge and membrane constriction followed by a scission event as the closing step.

The endosomal sorting complex required for transport (ESCRT) machinery has been linked to the process of abscission (Carlton and Martin-Serrano, 2007; Morita et al., 2007; Carlton et al., 2008; Guizetti and Gerlich, 2010). In addition to cytokinesis (Glotzer, 2001; Eggert et al., 2006; Barr and Gruneberg, 2007), the ESCRTs are also involved in a variety of other processes, such as multivesicular body (MVB) biogenesis (Teis et al., 2008; Tang et al., 2016), viral budding (Garrus et al., 2001) and nuclear membrane reformation (Stoten and Carlton, 2018). The common aspect of all these processes is that they are all related to membrane remodeling. The core of the ESCRT machinery consists of ESCRT-0, ESCRT-I, ESCRT-II, and ESCRT-III complexes and vacuolar protein sorting-associated protein 4 (VPS4) (Olmos and Carlton, 2016; Christ et al., 2017; Zhu et al., 2017; Gatta and Carlton, 2019). In eukaryotes, ESCRT-III and VPS4 are involved in the late phases of cytokinesis (Adell and Teis, 2011; Vietri et al., 2015). This is in contrast to the role of ESCRT-I and ESCRT-II, which coordinate the assembly of downstream complexes necessary for cytokinesis (Schmidt and Teis, 2012). Reporting on the role of the ESCRT-III complex and associated protein components, we provide here an structural view on abscission focusing mainly on mammalian cells.

## Formation of the Intercellular Bridge

The initiation of mitotic exit is based on cyclin-dependent kinase (CDK1), a universal protein kinase regulator. Cytokinesis is associated with down-regulation of CDK1 activity by phosphoregulation carried out by the coordinated action of kinases, counteracting phosphatases and association with cyclins (Hunt, 1991). Misregulation of CDK1 can prevent cytokinesis presumably because of its role in guiding and maintaining cleavage signals (Wheatley and Wang, 1996; Wheatley et al., 1997). Besides CDK1, Aurora B-dependent phosphorylation of the mitotic kinesin-like protein 1 (MKLP1) (Guse et al., 2005) is also essential for cytokinesis (Eggert et al., 2006). After furrow ingression both Aurora B and MKLP1 localize to the midbody, suggesting that the two proteins are involved subsequently in the regulation of abscission. Timing of abscission is regulated by Aurora B through phosphorylation of the human ESCRT-III subunit, charged multivesicular body protein 4C (CHMP4C) (Capalbo et al., 2012) in concert with VPS4 and abscission/NoCut checkpoint regulator (ANCHR) (Thoresen et al., 2014).

**FIGURE 1:**
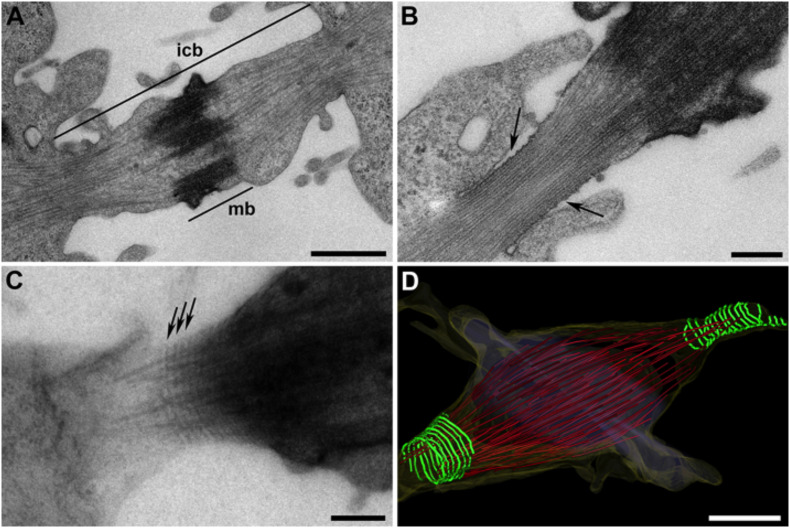
Ultrastructure of the abscission machinery in HeLa cells. **(A)** Thin-section electron micrograph of a chemically fixed intercellular bridge (icb). This overview image also shows the midbody (mb). **(B)** Visualization of a secondary constriction site. Image of a chemically fixed intercellular bridge showing a typical “ruffled zone” with membrane thickening (arrows). **(C)** Semi-thick serial section of a high-pressure frozen, freeze substituted and plastic-embedded sample as visualized by electron tomography. Filamentous structures (arrows) oriented perpendicular to the long axis of the bridge are shown. **(D)** 3D model of an intercellular bridge revealed by serial-section electron tomography. 3D rendering of ESCRT-III-dependent filaments (green), microtubules (red) and the cell membrane (yellow). The filaments show different degrees of constriction as previously reported (Guizetti et al., 2011). Scale bars **(A,D)**, 1 μm; **(B,C)**, 250 nm.

Concomitant with the rearrangement of the mitotic spindle into the central spindle, the actomyosin ring induces furrow ingression, thus leading to the formation of the intercellular bridge containing the midbody (Figure 1A). Actomyosin-driven contraction of the cleavage furrow continues until the intercellular bridge with the midbody at its center has been formed (Mierzwa and Gerlich, 2014). The ingressed furrow then needs to be stabilized to prevent furrow regression. Centralspindlin was identified as a protein complex with plasma membrane tethering activity. The C1 domain of the centralspindlin subunit, male germ cell rac GTPase-activating protein (MgcRacGAP) associates with phosphoinositide lipids (Lekomtsev et al., 2012). This link is thought to have a stabilizing role to secure the final steps of cytokinesis in animal cells. An additional protein with a stabilizing function of the midspindle is protein regulator of cytokinesis 1 (PRC1) (Lee et al., 2015). Centralspindlin and PRC1 show microtubule binding activities. Both proteins co-localize to the midbody and recruit additional factors, such as Kruppel-like factor (KLF4) (Lee et al., 2015). PRC1 is required for midzone formation, and KLF4 is necessary for organization of the central spindle (Kurasawa et al., 2004). The contractile ring protein, anillin was also proposed to play a role in stabilizing the membrane invagination after myosin II-mediated force production ceases (Field and Alberts, 1995). Furthermore, anillin recruits septins, involved in the formation of the constriction sites (Renshaw et al., 2014). The C-terminus of anillin can bind septins, linking anillin and septins to membrane stabilization (Kinoshita et al., 2002; Menon and Gaestel, 2015; Karasmanis et al., 2019).

## Models of Abscission

The physical separation of post-mitotic daughter cells is accompanied by a continuous decrease in the diameter of the intercellular bridge at the two opposing sides of the midbody. Initiated by furrow ingression, abscission is the net result of many complex and precisely orchestrated subprocesses (Agromayor and Martin-Serrano, 2013; Henne et al., 2013; Mierzwa and Gerlich, 2014; Frémont and Echard, 2018; Karasmanis et al., 2019). According to the vesicle-mediated model, midbody ring-localized secretory vesicles fuse with each other and with the plasma membrane. Release of the vesicle content is thought to be mediated by synaptosomal-associated protein 23/25 (SNAP23/25) and vesicle-associated snap receptor (v-SNARE) (Takahashi et al., 2004). Thus, abscission is proposed to take place at the site of vesicle fusion (Gromley et al., 2005). The caveat is that vesicles were no longer detectable within the intercellular bridge by the time abscission occured (Guizetti et al., 2011). In contrast to the vesicle-mediated model, the mechanical force model suggests that traction based forces generated by adherent cells induce abscission. Based on a comparison to the *Xenopus* oocyte, this model suggests a “wound-induced closure” mechanism at late cytokinesis (Darenfed and Mandato, 2005; Schiel and Prekeris, 2010).

In the following sections, we will focus on the role of ESCRT proteins and their spatial distribution in cell division. ESCRT-III components are primary candidates for the completion of cytokinesis (Wollert et al., 2009). Secondary constriction sites develop either at both sides of the midbody or in some cases only at one side (Mullins and Biesele, 1977; Schiel et al., 2012). In search of interacting partners of ESCRT-III, it was found that tumor susceptibility gene 101 protein (TSG101), a subunit of ESCRT-I, binds the centrosomal protein of 55 kDa (CEP55) (Fabbro et al., 2005) and the apoptosis-linked gene 2-interacting protein x (ALIX) (Morita et al., 2007; Carlton et al., 2008). In fact, CEP55 recruits TSG101 and ALIX to the midbody (Lee et al., 2008). Depletion of ALIX by RNA interference resulted in a 14-fold increase in multinucleated cells compared to control cells, suggesting a role for ALIX in cytokinesis (Carlton and Martin-Serrano, 2007). ALIX acts as an adaptor molecule connecting CEP55 with ESCRT-III to initiate completion of cytokinesis (Carlton et al., 2008). The CHMP4 subunits of ESCRT-III localize next to the midbody and polymerize toward the direction of the constriction site (Guizetti et al., 2011). In support of this observation, membrane deforming and interaction properties of ESCRT-III components were documented (Darenfed and Mandato, 2005; Hanson et al., 2008; Malerød and Stenmark, 2009). Based on studies of syndecan–syntenin–ALIX interaction in the case of exosome formation (Baietti et al., 2012), a recent study suggested a two-phase recruitment of ESCRT-III, involving a tripartite module (ALIX-syntenin-syndecan-4) to be essential for localization and abscission. ALIX-syntenin anchors ESCRT-III to the membrane, while syndecan-4 stabilizes ESCRT-III polymers at the abscission site (Addi et al., 2020).

Importantly, ESCRT-III–mediated secondary constriction requires synchronization with the disassembly of microtubules within the intercellular bridge. Increased concentration of the microtubule-severing enzyme, spastin can be measured at the site where microtubule disassembly takes place (Yang et al., 2008; Connell et al., 2009; Guizetti et al., 2011). How the components of the ESCRT-III machinery are orchestrated during abscission *in vivo* remains largely elusive. One possibility is that plasma membrane-attached ESCRT-III filaments increase their curvature as they extend from the midbody, constricting the membrane until scission takes place (Guizetti et al., 2011). Another possibility involves an increase in constriction by remodeling ESCRT-III subunits by Vps4 (Mierzwa et al., 2017). It has also been proposed that filaments may slide along the intercellular bridge and exert constriction determined by the elastic forces in the membrane (Elia et al., 2012).

## Structural Studies on Isolated ESCRT Components

The ESCRT machinery is conserved from archaea to humans (Hurley and Emr, 2006; Lindås et al., 2008; Schöneberg et al., 2017; Gupta et al., 2020). The 12 human homologs of ESCRT-III and VPS4 were shown to contribute to cytokinesis (Carlton and Martin-Serrano, 2007; Morita et al., 2007; Caballe and Martin-Serrano, 2011; Schöneberg et al., 2017). The ESCRT-III proteins show structural variance, but the four N-terminal helices are homologous. The C-terminal truncated CHMP38–183 crystal structure with a truncated C-terminal acidic domain (Muzioł et al., 2006) served as a search model for molecular replacement of CHMP38–222 (Bajorek et al., 2009).

Depending on the composition of the lipid monolayer and the interacting ESCRT-III subunits, self-assembled ESCRT-III heteropolymers can adopt structures of variable dynamics and architectures such as rings, spirals, helices or linear polymers (Hanson et al., 2008; Henne et al., 2012). As an example, yeast vacuolar-sorting protein (Snf7^CHMP4A;B;C^), a core component of ESCRT-III polymerizes into spirals at the surface of lipid bilayers, mediating membrane deformation and fission (Chiaruttini et al., 2015). In addition the ESCRT-III subunits isolated from yeast, Vps2^CHMP2A,B^, Vps20^CHMP6^, Vps24^CHMP3^, and also Snf7 were proposed to form filaments and deform membranes (Cashikar et al., 2014). Although Snf7 by itself is unable to induce buckling on artificial lipid membranes, Vps2/Vps24 drive the formation of spirals (Henne et al., 2012) and form a parallel strand through electrostatic interaction with Snf7 (Mierzwa et al., 2017; Mandal et al., 2020). Incubation of Snf7/Vps2/Vps24 with detergent-solubilized lipids resulted in helices of different forms (Moser von Filseck et al., 2020).

A recent cryo-electron microscopy (cryo-EM) study at 3.2 Å resolution showed a double-stranded helical arrangement of Vps24. Lipids were shown to assist in the disassembly of the filaments. This study further revealed the following three important aspects of Vps24 function: First, apolar Vps24 integrates into linear polar Snf7 polymers, thereby neutralizing the polarity so that opposite polarity filaments can combine (Huber et al., 2020). Second, it was shown that Vps24 has an intrinsical helical property. Under defined conditions, Vps24 is capable of inducing curvature in flat Snf7 polymers (Henne et al., 2012; Tang et al., 2015). Third, Vps24 consists of two protofilaments. Based on a lateral association of Vps2/Vps24 and Snf7, this could allow a combination of multiple linear Snf7 filaments (Mierzwa et al., 2017; Huber et al., 2020). All this suggested that Vps24 is a candidate for an adaptor complex that connects ESCRT-III homopolymers (Huber et al., 2020).

In a study of ESCRT-III-driven shaping of positively curved membranes, the membrane-bound complex CHMP1B-IST1 was shown to constrict the underlying membrane bilayer nearly to the fission point by a two-component sequential polymerization mechanism (Nguyen et al., 2020). Spontaneous co-assembly between CHMP1 and truncated IST1 frequently resulted in conical helix shapes with decreasing diameter under low ionic strength, whereas an increasing ionic strength favored the monomeric form of the subunits (McCullough et al., 2015). The other way round, membrane shape was also reported to influence the assembly of ESCRT-III subunits. In contrast to *in vivo* conditions, *in vitro* experiments showed that CHMP2B and CHMP2A/CHMP3 assemble on positively curved but not on negatively curved membranes. This suggests that these complexes don’t assemble inside membrane structures (negative curvature) but rather on the outside surface (positive curvature). Moreover, CHMP4B has a preference to bind either to flat membranes or to tubes with positive mean curvature (Bertin et al., 2020). Last but not least, it is important to note that polymeric structures can also form even in the absence of membranes (Henne et al., 2012; Banjade et al., 2019). Therefore, it will be crucial in future studies to verify the reported molecular shapes and membrane interactions of the various ESCRT-III components within the cellular context of abscission.

## Role of VPS4 in Regulating ESCRT-III

Vps4 is a member of the meiotic clade AAA-ATPase and a key component of the ESCRT pathway (Monroe and Hill, 2016). Its N-terminus consists of a microtubule interacting and trafficking (MIT) domain, which interacts with carboxy-terminal peptide, MIT interaction motifs (MIM) in ESCRT-III proteins (Obita et al., 2007; Stuchell-Brereton et al., 2007; Kieffer et al., 2008). This is followed by a linker region (Shestakova et al., 2013) and an AAA-ATPase cassette that consists of a large ATPase and a small ATPase domain, a beta domain and a C-terminal helix (Scott et al., 2005; Gonciarz et al., 2008). Vps4 also binds the activator vacuolar protein sorting-associated protein (Vta1) (Yeo et al., 2003; Shestakova et al., 2010), which contacts the beta domain and regulates assembly of Vps4 and ATPase activity (Azmi et al., 2006). In the cytosol, Vps4 is present mainly as a monomer but it forms hexamers in its activated form (Monroe et al., 2014; Adell et al., 2017). The hexamers are associated to each ESCRT-III filament by the MIT-MIM interaction. The cryo-EM structure at 4.3 Å resolution revealed that Vps4 is highly asymmetric and stabilized by ATP and Vta1 binding. One end of the Vps4 molecule grows and hydrolyzes ATP, while the other end disassembles (Monroe et al., 2017). The disassembled subunits were reported to move to the growing site, creating a “walking” motion of Vps4 along the ESCRT-III filament, pulling it through the pore of the hexamer and therefore disassembling the filament (Yang et al., 2015; Adell et al., 2017; Monroe et al., 2017).

While regulating assembly and disassembly of ESCRT-III complexes, Vps4 can also promote shape adaptations to membranes with different curvatures (Mierzwa et al., 2017), allowing Vps4 to function in a wide range of processes. High-speed atomic force microscopy (HS-AFM) imaging of Vps4 with Vps2, Vps24, and Snf7 on lipid bilayers showed ESCRT-III spirals with reduced diameter in the presence of ATP. From this observation it was concluded that Vps4 is responsible for a dynamic subunit turnover and that Vps2, Vps24 subunits function as inhibitors (Mierzwa et al., 2017). Vps24 induced curvature when mixed with flat Snf7 filament polymers (Henne et al., 2012; Tang et al., 2015). Vps2/Vps24 subunits of ESCRT-III formed side by side filaments with Snf7 (Mierzwa et al., 2017). Furthermore, Vps2/Vps24 is crucial for Vps4–mediated remodeling. Polymerized Snf7 on membranes mixed with Vps4 and ATP didn’t induce remodeling of the Snf7 filaments. However, the combination of Snf7, Vps2/Vps24, Vps4, and ATP resulted in both shrinking and growth of ESCRT-III spirals (Mierzwa et al., 2017). This dynamic remodeling of ESCRT-III argues against the previously proposed “cut and slide” model, which suggested a single cut by VPS4 to trigger constriction (Elia et al., 2012).

## Structural Approaches to Study the Function of ESCRTs *in vivo*

Structural analysis of cytokinesis is challenging given that the spatial organization of the abscission machinery is complex and the interactions of the different components are dynamic and possibly transient. In order to reduce the complexity, cytokinesis has been studied predominantly under *in vitro* conditions. This approach allows the assessment of the roles of specific components of the machinery by taking them out of the physiological context and placing them into a defined artificial environment. As a result of the “resolution revolution”, cryo-EM is now the method of choice for the determination of 3D structures of large macromolecular assemblies *in vitro* at near atomic resolution (Callaway, 2020). Ultimately, however, cytokinesis should also be studied within the cellular context.

The first *in vivo* study on cytokinesis used fixed HeLa cells and revealed an elongated intercellular bridge (Byers and Abramson, 1968). The elongated bridge showed membrane thickenings, called “waves.” These waves were claimed to relate to translational forces acting along arrays of microtubules. Concerted waves passing from the midbody to the cell were thought to exert forces on the connected cells, resulting in a separation of the cell pair (Byers and Abramson, 1968). Using D-98S cells, it was possible to quantify the change in the diameter of the intercellular bridge over time. The reduction in diameter was attributed to two subprocesses (Mullins and Biesele, 1977): first, a systematic breakdown of the midbody structure, and second a constriction causing a disintegration and a spatial rearrangement of microtubules. Along the narrowed plasma membrane of cellular bridges, wave-like ripples on both sides adjacent to the midbody were also observed in this experiment analyzing chemically fixed cells (see also Figure 1B for an example in HeLa cells). However, there was no evident sub-cellular structure visible by thin-section electron microscopy causing these ripples. Stretching of the intercellular bridge was hypothesized to cause the final cut by reaching a “point of breakage” (Mullins and Biesele, 1977).

Membrane ripples were later observed by serial-section electron tomography of high-pressure frozen and freeze substituted intercellular bridges in HeLa cells (Guizetti et al., 2011). This study revealed intertwined helical filaments causing cortical constriction. The visualized filaments at the secondary constriction sites were 17.3 ± 2.5 nm wide and showed a helical pitch of 35.3 ± 4.1 nm (Figures 1C,D). Based on the helical properties of filaments assembled *in vitro* (Ghazi-Tabatabai et al., 2008; Hanson et al., 2008; Lata et al., 2008) and by comparison of wild-type and CHMP2A-depleted HeLa cells (Hanson et al., 2008; Guizetti et al., 2011), it was suggested that the *in vivo* observed filaments are polymerized ESCRT-III core components (Guizetti et al., 2011). However, a direct labeling of these ESCRT-III-dependent filaments, for instance by immuno-EM, has not been reported so far.

In terms of labeling, the antibody-stained ESCRT-III-subunit IST1 was visualized by stochastic optical reconstruction microscopy (STORM). It was shown that rings emanate parallel to the dark zone of the midbody. In later stages of abscission, spirals of IST1 were extending toward the abscission site with decreasing diameter and width (Goliand et al., 2018). Structural analysis of Madin-Darby canine kidney cells (MDCK) by soft X-ray cryo-tomography (Sherman et al., 2016) confirmed previous results regarding the membrane bulging from the midbody (Elia et al., 2011; Guizetti et al., 2011; Schiel et al., 2011; Crowell et al., 2014) and also showed membrane extrusions from the vicinity of the pre-abscission site. Three cortical rings were visualized with an average diameter of 1.43 ± 0.25 μm. Furthermore, three intertwined helices were imaged with an average diameter of 556 ± 15 nm, one of the helices extended to the abscission site (Sherman et al., 2016).

## Outlook

A number of *in vitro* ultrastructural studies of ESCRT-III components are based on single-particle analyses by cryo-EM (Su et al., 2017; Maity et al., 2019; Bertin et al., 2020; Huber et al., 2020; Moser von Filseck et al., 2020; Nguyen et al., 2020; Pfitzner et al., 2020). While *in vitro* experiments provide useful structural information about the building blocks of the abscission machinery under artificial conditions, it is difficult to correlate this information with the dynamic attributes of the cytokinetic apparatus under physiological conditions. In addition, isolated components may form artificial structures, thus leading to molecular mechanisms that may not fully represent the molecular processes as observed within cells. Along this line, *in vitro* purification can cause aggregation of ESCRT-III proteins (Wollert et al., 2009), induce dramatic conformational changes, diverse morphologies (Schöneberg et al., 2017) and non-physiological interactions of interdependent subunits. Furthermore, another problematic issue of *in vitro* studies is the lack of biomimetic substrates that capture the *in vivo* conditions on which ESCRT-III components can act upon. For all these reasons, an *in vitro* reconstruction of the abscission machinery might therefore be insufficient to explain the complexity of abscission within cells. Moreover, cellular crowding and local concentrations of the ESCRT-III components may have significant effects on the mechanism of abscission, inasmuch as time-dependent aspects need to be considered when analyzing the cytokinetic apparatus.

Interestingly, the number of *in vivo* ultrastructural analyses on cytokinesis is currently very small (Elia et al., 2011; Guizetti et al., 2011; Sherman et al., 2016; Mierzwa et al., 2017; Goliand et al., 2018). In fact, a systematic time-resolved study on the ultrastructure of abscission has never been performed. In addition, models on ESCRT-III function deduced from *in vitro* experiments need to be benchmarked against cellular conditions. Certainly, more *in vivo* structural studies would result in a better understanding of the abscission machinery in cells and clarify some fundamental questions such as: How does the geometry of ESCRT-III change during the elongation of the intercellular bridge? What are the interacting partners of ESCRT-III? How do they interact and coordinate with each other during cytokinesis? How do other cytoskeletal components cooperate with ESCRT-III proteins and how does RNAi silencing of helix forming subunits affect the ultrastructure of the abscission machinery? Moreover, how are the 17-nm cortical filaments anchored to the plasma membrane? To answer these questions, additional 3D analyses of intercellular bridges at defined time points need to be performed. Such time-resolved 4D studies will give important information on the development of the abscission machinery and the process of constriction.

In summary, while many high-resolution structures of subunits involved in abscission were solved under *in vitro* conditions, very little is known about the spatial organization and function of these subunits inside the cell. Therefore, *in vivo* ultrastructural studies should play an essential role in uncovering the discrete time-dependent steps of late cytokinesis. Subsequently, high-resolution structures should then be fitted into the electron density map of the abscission machinery. Thus, both *in vitro* and *in vivo* studies should be performed in parallel to grasp the full complexity of abscission across different imaging scales.

## Author Contributions

All authors listed have made a substantial, direct and intellectual contribution to the work, and approved it for publication.

## Conflict of Interest

The authors declare that the research was conducted in the absence of any commercial or financial relationships that could be construed as a potential conflict of interest.
